# Influence of Drugs, &c.

**Published:** 1887-10

**Authors:** 


### INFLUENCE OF DRUGS GIVEN TO THE MOTHER ON THE NURSING INFANT.

Experiments made by Dr. Fehling and reported in *Ther. Gaz.*, show that the administration of opium, morphine, chloral, iodoform and ferrocyanide of potassium to the nurse or mother, has no effect whatever upon the child. Chemical examinations of the excretions of the mother indicated the elimination of the drugs, but no evidence of their presence was found in the excretions of the child, nor did any symptoms present themselves in the child which would indicate that the drugs had effected it.

Mercury very slightly and inconstantly effected the child.

The administration of the salicylate of sodium in doses varying from thirty to forty-five grains, made an impression upon the child, if it were put to the breast within an hour—the drug manifesting its presence in the child's urine and disappearing within twenty-four hours, the elimination of the drug terminating simultaneously in mother and child.

The same results were obtained in the case of iodide of potassium.

Sulphate of atropine, of all drugs observed, produce the most prompt, positive, profound and prolonged effects upon the child, when administered to the mother or nurse, indicating that minute doses only of this drug are permissable.

This experiment gives further evidence of the supreme susceptibility of infants to atropine.

Dr. Fehling testifies that the influence of the nurse's diet on the child is illusory ; the nurse or mother can, with impunity, eat sour articles, such as lemons, oranges and vinegar, without thereby influencing the child.

How many mothers have, for days and weeks after their confinement, been denied satisfaction to their cravings for lemons, fruit and salads, by the Sarah Gramps ruling over them in their times of trial, the attending surgeons calmly, cruelly consenting, on account of the imaginary danger of colic to the puling infant —*Med. Sum.*

---

The New York College of Magnetics commences its fall term October 11th, at the ample lecture rooms of the College Building, 39 West 27th street, New York City. Some members of the late graduating class have already established themselves as active practitioners, under the system of healing introduced by Dean Prof. E. D. Babbitt, and are meeting with great success, in the cure of a class of diseases which have defied ordinary methods.

